# Supplementary material for: Replication dynamics of recombination-dependent replication forks
Source: Nat Commun. 2021 Feb 10;12:923. doi: 10.1038/s41467-021-21198-0 (PMC7876095; doi:10.1038/s41467-021-21198-0)
Supplement: Supplementary file 1 — Supplementary Information [file 41467_2021_21198_MOESM1_ESM.pdf]

Replication dynamics of recombination-dependent replication forks

**Supplementary Figure legends**

Supplementary Figure 1
**Polymerase usage following HR-restart at 4 different loci.** Pu-seq traces at three loci on ChrI and one locus on ChrII where the *RTS1* barrier has been integrated close to a strong origin in an early replicating region of the chromosome that boarders a late replicating region. *RTS1* barrier activity on (*rtf1*$^{+}$). The usage of Pol δ (blue) and Pol ε (red) are shown on the Watson and Crick strands. Note the switch from Pol ε to Pol δ at the *RTS1* site that is indicative of a change in polymerase usage from Pol ε to Pol δ on the leading strand when *RTS1* barrier activity is on and replication is restarted by HR.

Supplementary Figure 2
**Calculating the delay to replication at rRFB barrier.** Pu-seq traces of a locus on ChrI with and without the integration of 10xTer2-Ter3 sequence (rRFB barrier). **a**. Top Panel: Fork direction calculated from the Pu-seq traces (see[17]). When the rRFB barrier construct is integrated, there is a decrease in the proportion of left-right moving forks downstream of the barrier. This is caused by a delay to left-right moving forks, and a concomitant increase in right-left moving forks. Bottom panel. Polymerase delta usage averaged across both strands is shown, demonstrating that (unlike for *RTS1*) forks only pause and are not restarted by HR. **b**. Selected outputs (4 - 8 mins, 1 minute intervals) of the Monte Carlo model for the indicated delays to the left-right replication forks at rRFB are compared to the experimental data. **C**. Error between the experimental data and the model output for a delay between 0 and 8 minutes (15 second intervals) measured by the Euclidean distance between the two signals; orange curve is a smoothed representation of the errors using a Savitzky-Golay filter.

Supplementary Figure 3
**Calculating the delay at *RTS1* barrier.** Using the replication model, different delay times of replication at the *RTS1* barrier before HR-dependent restart (between 0 and 30 minutes, 1 minute steps) were fitted and compared to the experimental data. The best fit is an 11 minute delay at the barrier. **a**. Selected outputs from the model for usage of Pol δ on Watson strand are compared to the experimental data. Note: both when replicated by left-right HR restarted forks, or when replicated by right-left converging canonical forks, the Watson strand is replicated by Pol δ. **b**. Selected outputs from the model for usage of Pol δ on Crick strand are compared to the experimental data. Note: A decrease in Pol δ usage on the Crick strand reflects replication by Pol ε from right-left converging forks. Thus, because an increased delay results in more replication by converging canonical forks, this is reflected by a decrease in Pol δ usage. **c**. Error between the acquired data and the model output measured by the Euclidean distance between the two signals; orange curve is a smoothed representation of the errors using a Savitzky-Golay filter.

Supplementary Figure 4
**Comparative polymerase usage following HR-restart between *rnh201-d* and *rnh201-RED*.** Pu-seq traces of the *RTS1-rRFB* locus on ChrII. Top two traces: *RTS1* barrier activity off (*rts1-d*). Bottom two traces: *RTS1* barrier activity on (*rtf1*$^{+}$). The usage of Pol δ (blue), Pol ε (red), and Pol α (green) are shown on the Watson and Crick strands in *rnh201-d* and *rnh201-RED*.

Supplementary Figure 5

**Efficiency of the *RTS1* barrier depends on the expression level of *rtf1⁺*. a**. The endogenous *rtf1*$^+$ promoter results in 70% barrier efficiency. Left: Selected outputs of the model for different efficiencies of delay are compared to the experimental data. Right: Error between the experimental data and the model output (5% steps) measured by the Euclidean distance between the two signals. **b**. adh1-*rtf1* constitutive overexpression results in 90% barrier efficiency. Left: Selected outputs of the model for different efficiencies of delay compared to the experimental data. Right: Error between the experimental data and the model output (5% steps) measured by the Euclidean distance between the two signals.

Supplementary Figure 6

**Delay at second *RTS1* barrier after HR restart at the first *RTS1*.** Using the replication model, different delays for replication of HR-restarted forks at the second *RTS1* barrier (between 0 and 30 minutes, 1 minute steps) were fitted to the experimental data. **a**. Selected outputs from the model for usage of Pol δ on the Watson strand compared to the experimental data. Note: both when replicated by left-right HR restarted forks, or when replicated by right-left converging canonical forks, the Watson strand is replicated by Pol δ. **b**. Selected outputs from the model for usage of Pol δ on Crick strand compared to the experimental data. Note: A decrease in Pol δ usage on the Crick strand reflects replication by Pol ε from right-left converging forks. Thus, because an increased delay results in more replication by converging canonical forks, this is reflected by a decrease in Pol δ usage. **c**. Error between the experimental data and the model output measured by the Euclidean distance between the two signals; orange curve is a smoothed representation of the errors using a Savitzky-Golay filter.

Supplementary Figure 7

**Construction and testing of *rnh201-RED*.** In *S. cerevisiae* it has been shown that the synthetic growth defect between the combined *rnh201* and *rnh1* deletions with deletion of *TOP1* is alleviated by restoration of the poly-ribonuclease activity of Rnh201 (i.e. *rnh201-RED*). **a**. Alignment of *S. cerevisiae* and *S. pombe* Rnh201 with highlighted amino acid changes. **b**. Relative DNA fragmentation at incorporated rNTPs after alkaline treatment (for details see the methods section). **c**. Spot test: *top1-d rnh1-d rnh201-d* grow slower than the wild type control (*rnh201*$^+$) and *top1-d rnh1-d rnh201-RED*.

Supplementary Figure 8

**Delay at *RTS1* before HR-restart in absence of Pku70.** Using the replication model different delay times of replication at the *RTS1* barrier before HR-dependent restart for the *pku70-d* background were fitted (30 minutes, 1 minute intervals) and compared to the experimental data. The best fit is a 14 minute delay. **a**. Selected outputs from the model for usage of Pol δ on Watson strand compared to the experimental data. Left panel: *pku70-d rnh201-d*. Right panel: *pku70-d rnh201-RED*. Note: both when replicated by left-right HR restarted forks, or when replicated by right-left converging canonical forks, the Watson strand is replicated by Pol δ. **b**. Selected outputs from the model for usage of Pol δ on Crick strand compared to the experimental data. Left panel: *pku70-d rnh201-d*. Right panel: *pku70-d rnh201-RED*. Note: A decrease in Pol δ usage on the Crick strand reflects replication by Pol ε from right-left converging forks. Thus, because an increased delay results in more replication by converging canonical forks, this is reflected by a decrease in Pol δ usage. **c**.

Error between the experimental data and the model output measured by the Euclidean distance between the two signals; orange curve is a smoothed representation of the errors using a Savitzky-Golay filter. Left panel: *pku70-d rnh201-d*. Right panel: *pku70-d rnh201-RED*.

Supplementary Figure 9
**Position of replication restart in the absence of Pku70.** Using the replication model, different start points of HR-dependent replication restart were fitted with 100 base steps downstream or upstream and compared to the experimental data. **a**. Selected outputs from the model for usage of Pol δ on Watson strand compared to the experimental data. Note that a 100 bp change in the position of restart shifts the transition of Pol δ usage. **b**. Selected outputs from the model for usage of Pol δ on Crick strand compared to the experimental data. **c**. Error between the experimental data and the model output measured by the Euclidean distance between the two signals; orange curve is a smoothed representation of the errors using a Savitzky-Golay filter. For this plot the primary sequence data was reanalysed (Bowtie2) with a bin size of 100 and the bin sizes in the model adjusted accordingly. The experimental data becomes more noisy as bin size deceases, and thus the experimental data is smoothed with a sliding window of 5.

**Fig.S1**

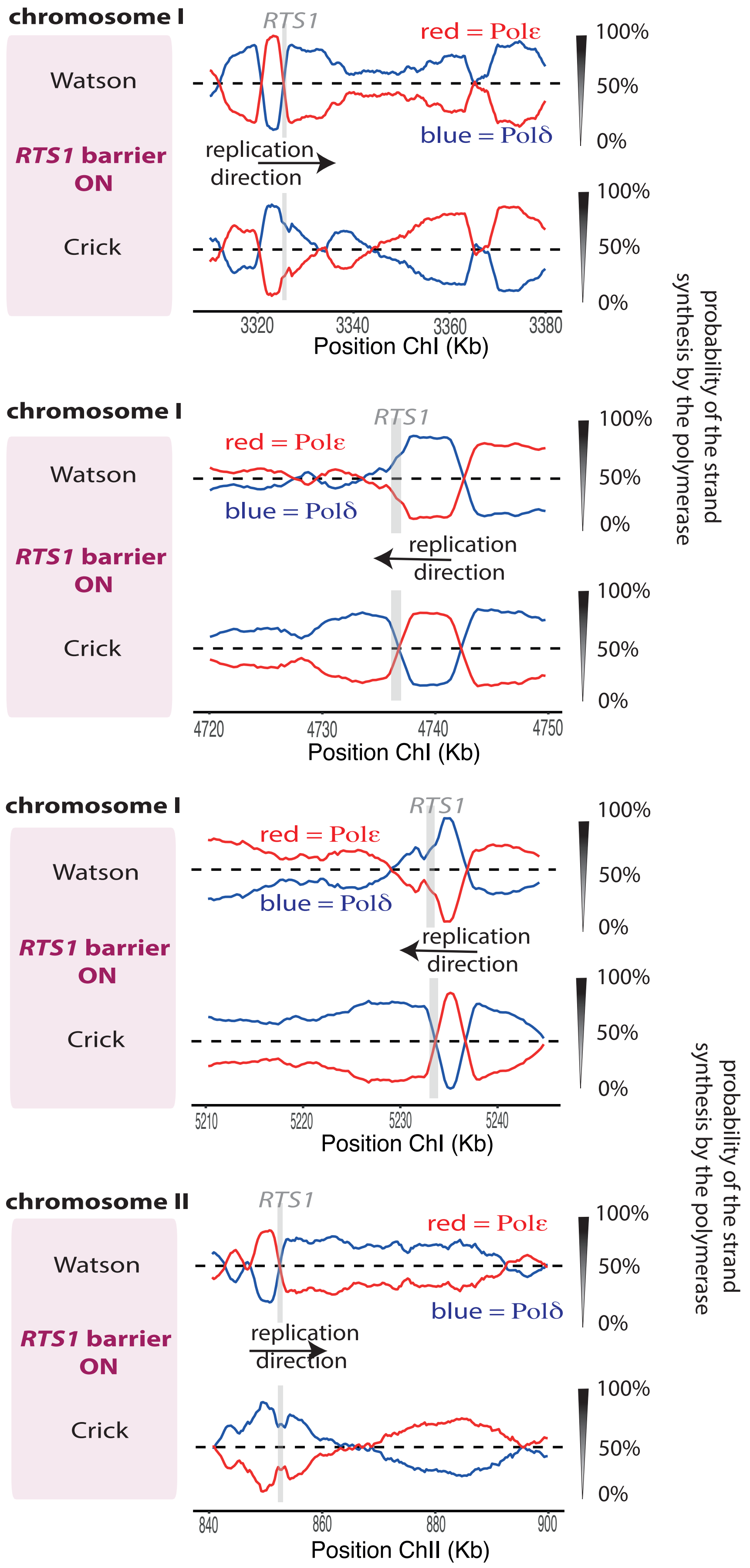
chromosome I
Watson
RTS1 barrier ON
Crick
RTS1
red = Polε
blue = Polδ
replication direction
100%
50%
0%
3320
3340
3360
3380
Position ChI (Kb)
probability of the strand synthesis by the polymerase
4720
4730
4740
4750
5210
5220
5230
5240
chromosome II
840
860
880
900
Position ChII (Kb)

**Fig.S2**

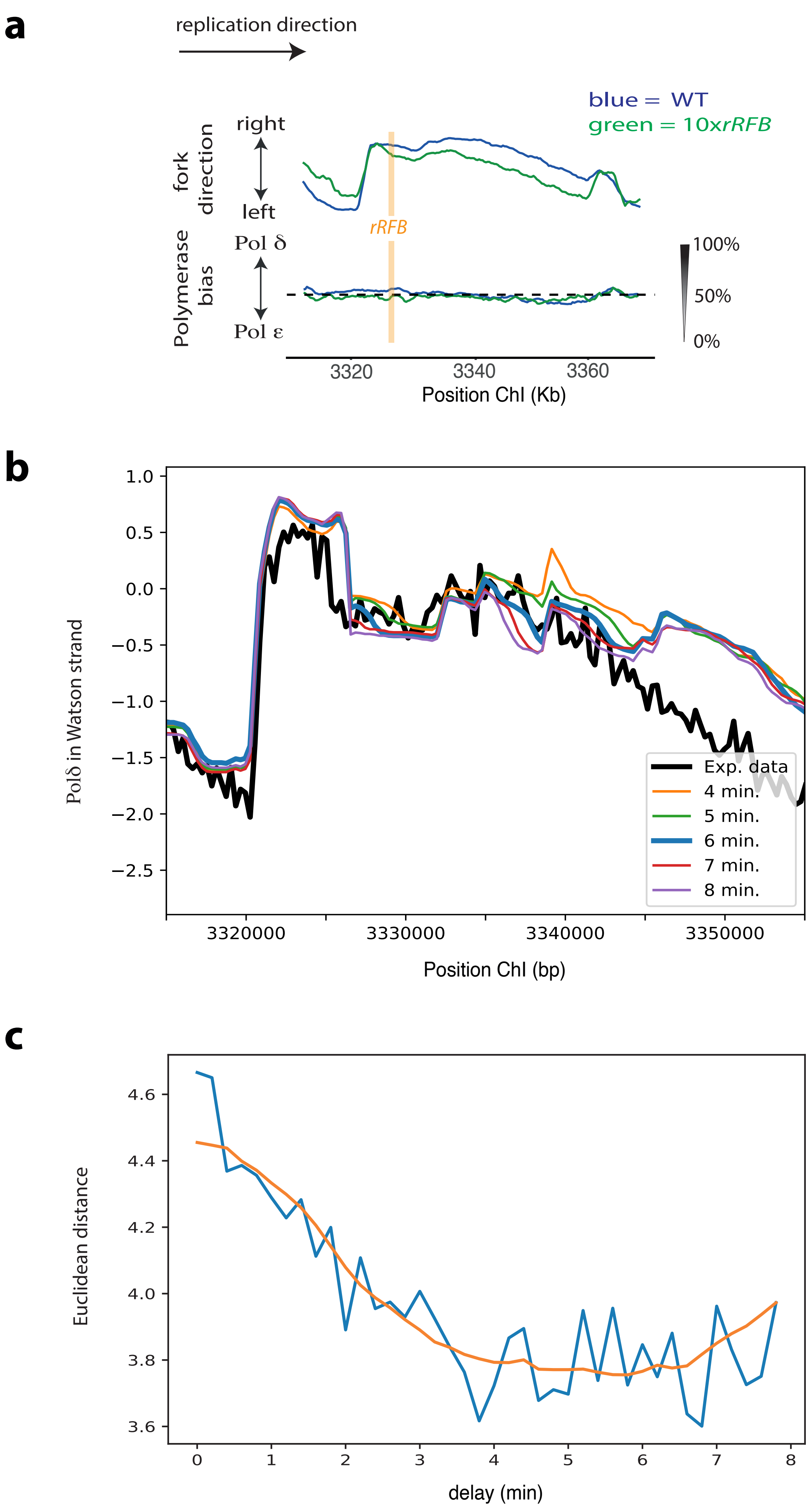
a
replication direction
blue = WT
green = 10xrRFB
right
left
fork direction
rRFB
Pol δ
Pol ε
Polymerase bias
100%
50%
0%
3320
3340
3360
Position ChI (Kb)
b
1.0
0.5
0.0
−0.5
−1.0
−1.5
−2.0
−2.5
Polδ in Watson strand
Exp. data
4 min.
5 min.
6 min.
7 min.
8 min.
3320000
3330000
3340000
3350000
Position ChI (bp)
c
4.6
4.4
4.2
4.0
3.8
3.6
Euclidean distance
0
1
2
3
4
5
6
7
8
delay (min)

Fig.S3

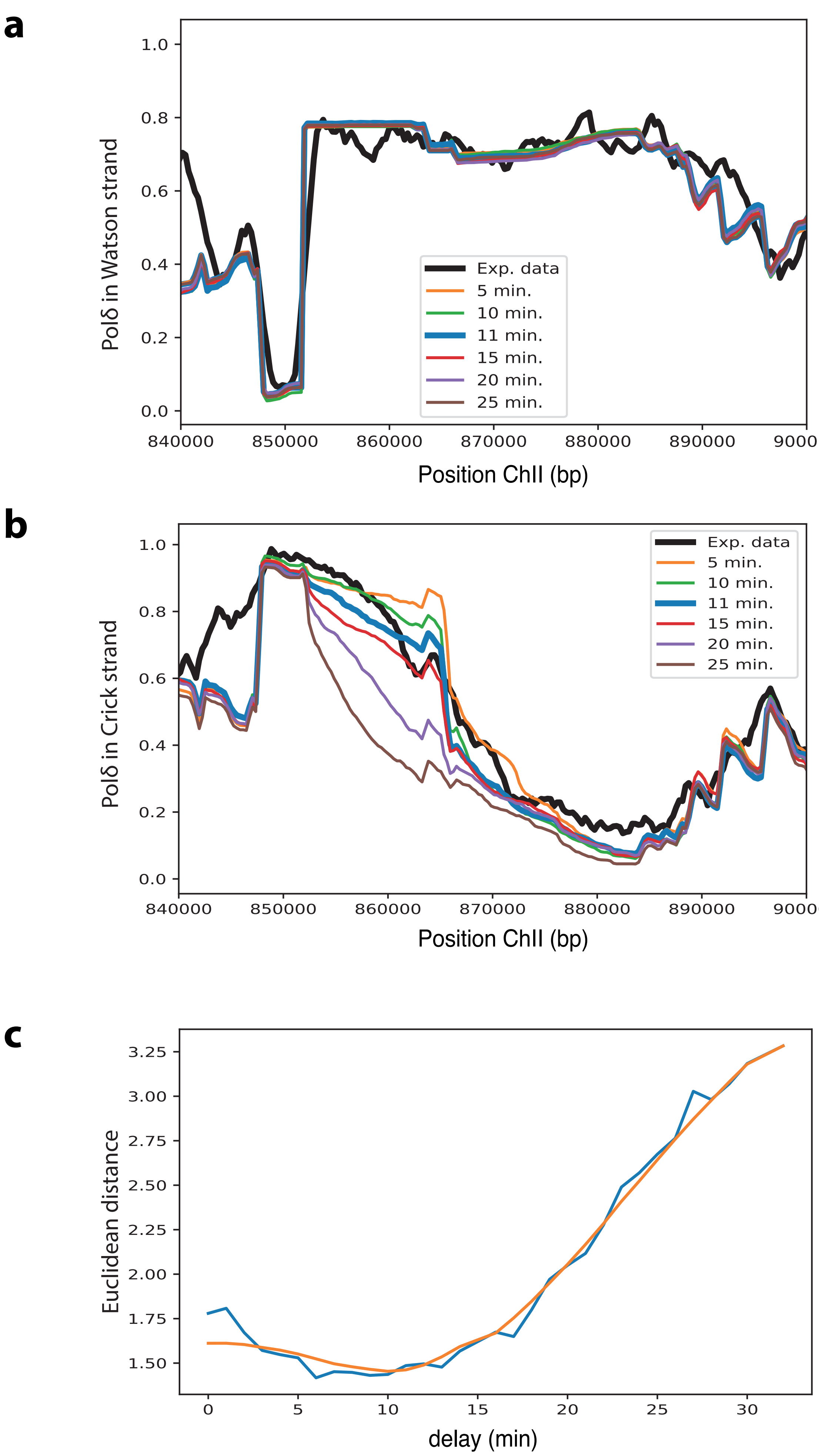
a
Polδ in Watson strand
1.0
0.8
0.6
0.4
0.2
0.0
840000
850000
860000
870000
880000
890000
900000
Position ChII (bp)
Exp. data
5 min.
10 min.
11 min.
15 min.
20 min.
25 min.
b
Polδ in Crick strand
Position ChII (bp)
c
Euclidean distance
3.25
3.00
2.75
2.50
2.25
2.00
1.75
1.50
0
5
10
15
20
25
30
delay (min)

**Fig.S4**

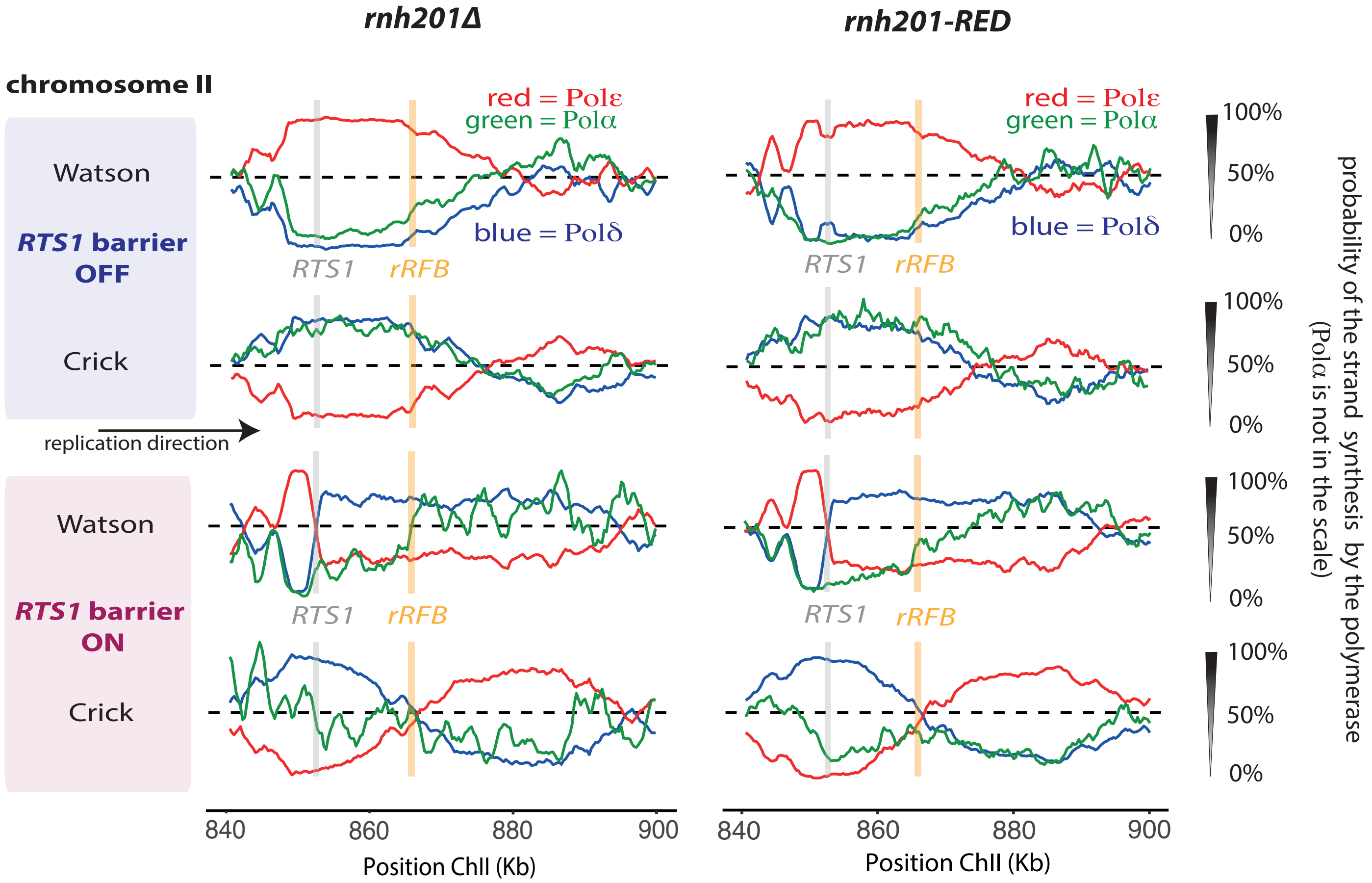
rnh201Δ
rnh201-RED
chromosome II
red = Polε
green = Polα
blue = Polδ
Watson
RTS1 barrier OFF
Crick
replication direction
Watson
RTS1 barrier ON
Crick
RTS1
rRFB
100%
50%
0%
probability of the strand synthesis by the polymerase
(Polα is not in the scale)
840
860
880
900
Position ChII (Kb)

**Fig.S5**

**a** *rtf1+*

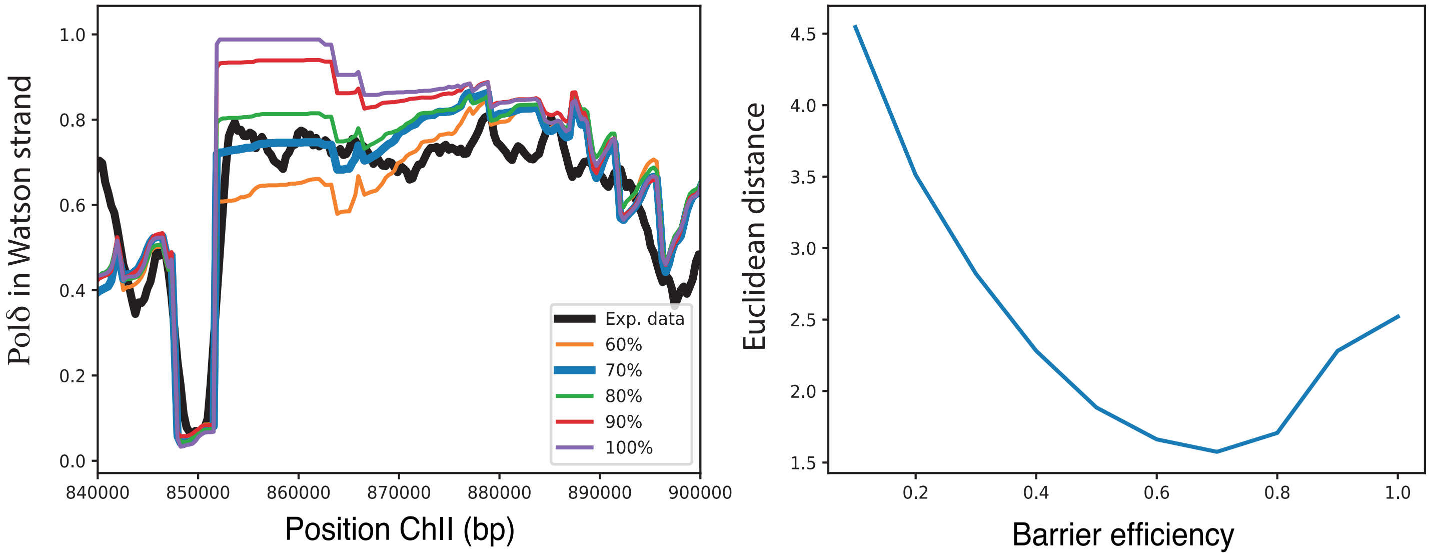


**b** *adh-rtf1*

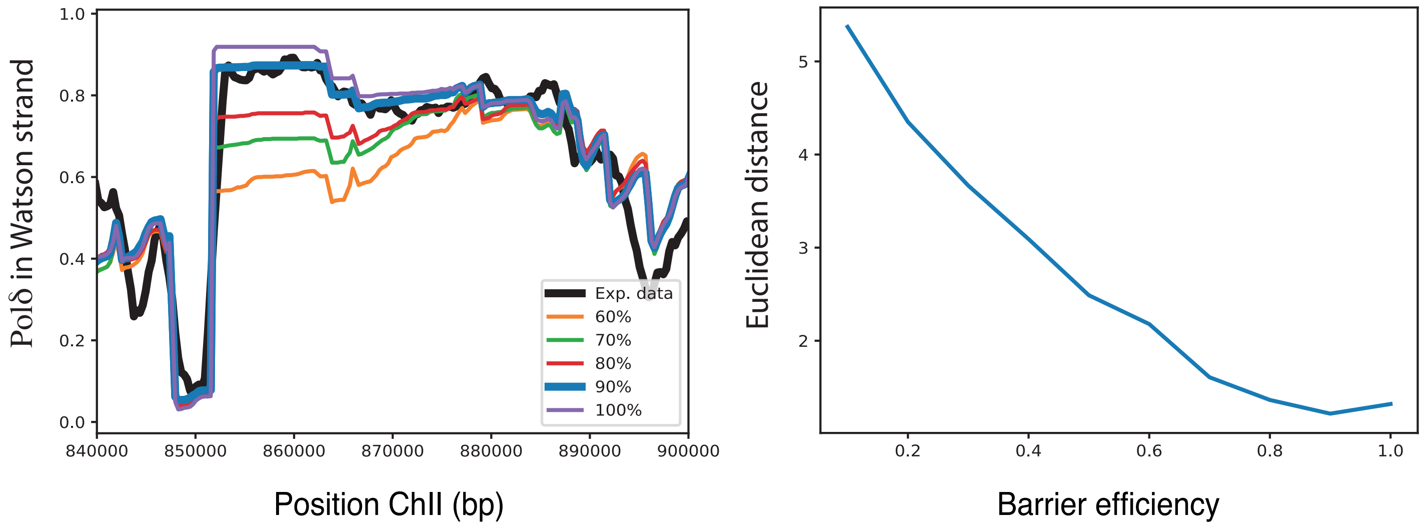

Fig.S6

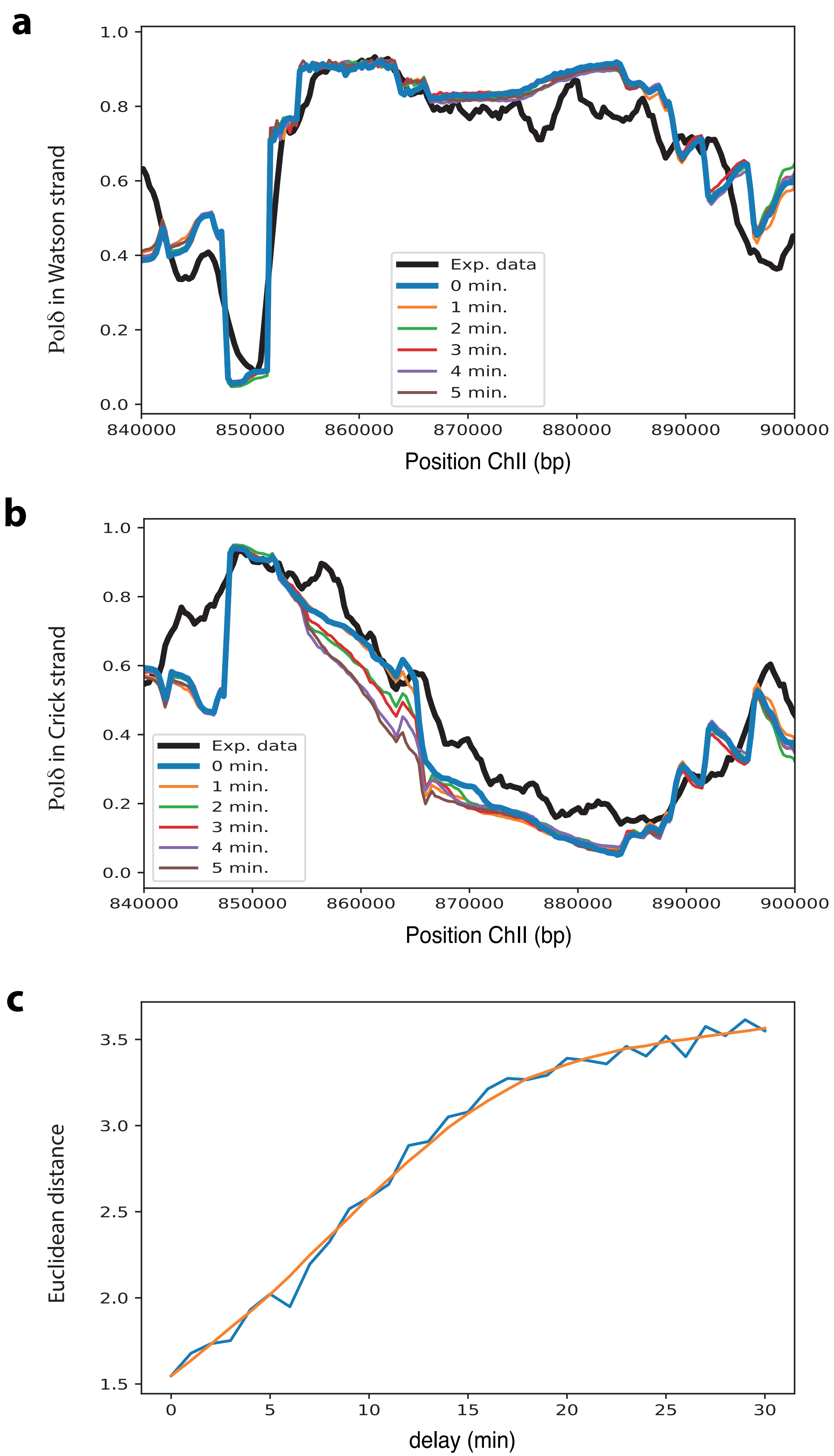
a
1.0
0.8
0.6
0.4
0.2
0.0
Polδ in Watson strand
840000
850000
860000
870000
880000
890000
900000
Position ChII (bp)
Exp. data
0 min.
1 min.
2 min.
3 min.
4 min.
5 min.
b
Polδ in Crick strand
Position ChII (bp)
c
3.5
3.0
2.5
2.0
1.5
Euclidean distance
0
5
10
15
20
25
30
delay (min)

**Fig S7**

**a**

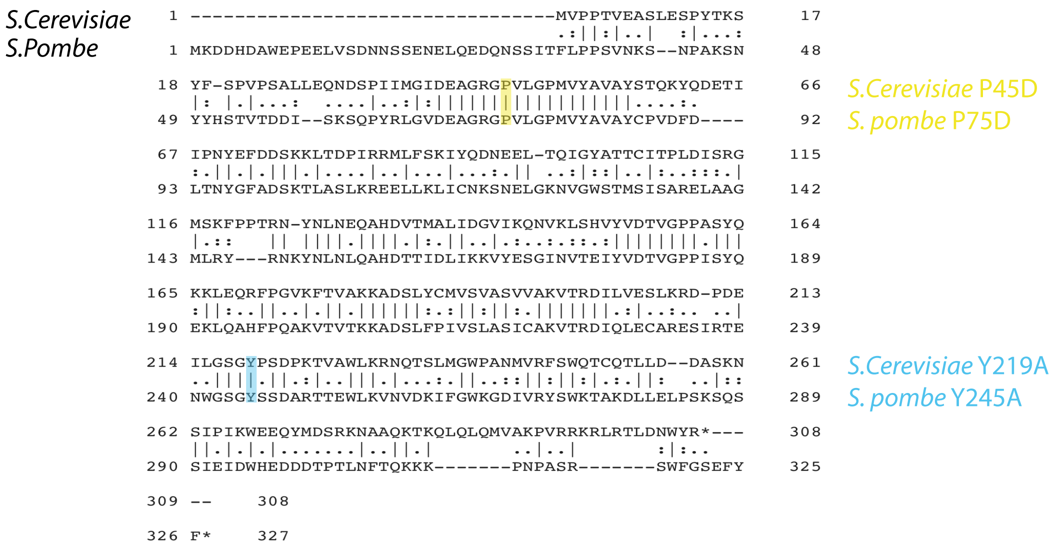
S.Cerevisiae
S.Pombe
1 ---------------------------------MVPPTVEASLESPYTKS 17
1 MKDDHDAWEPEELVSDNNSSENELQEDQNSSITFLPPSVNKS--NPAKSN 48
18 YF-SPVPSALLEQNDSPIIMGIDEAGRGPVLGPMVYAVAYSTQKYQDETI 66
49 YYHSTVTDDI--SKSQPYRLGVDEAGRGPVLGPMVYAVAYCPVDFD---- 92
67 IPNYEFDDSKKLTDPIRRMLFSKIYQDNEEL-TQIGYATTCITPLDISRG 115
93 LTNYGFADSKTLASLKREELLKLICNKSNELGKNVGWSTMSISARELAAG 142
116 MSKFPPTRN-YNLNEQAHDVTMALIDGVIKQNVKLSHVYVDTVGPPASYQ 164
143 MLRY---RNKYNLNLQAHDTTIDLIKKVYESGINVTEIYVDTVGPPISYQ 189
165 KKLEQRFPGVKFTVAKKADSLYCMVSVASVVAKVTRDILVESLKRD-PDE 213
190 EKLQAHFPQAKVTVTKKADSLFPIVSLASICAKVTRDIQLECARESIRTE 239
214 ILGSGYPSDPKTVAWLKRNQTSLMGWPANMVRFSWQTCQTLLD--DASKN 261
240 NWGSGYSSDARTTEWLKVNVDKIFGWKGDIVRYSWKTAKDLLELPSKSQS 289
262 SIPIKWEEQYMDSRKNAAQKTKQLQLQMVAKPVRRKRLRTLDNWYR*--- 308
290 SIEIDWHEDDDTPTLNFTQKKK-------PNPASR-------SWFGSEFY 325
309 -- 308
326 F* 327
S.Cerevisiae P45D
S. pombe P75D
S.Cerevisiae Y219A
S. pombe Y245A

**b**

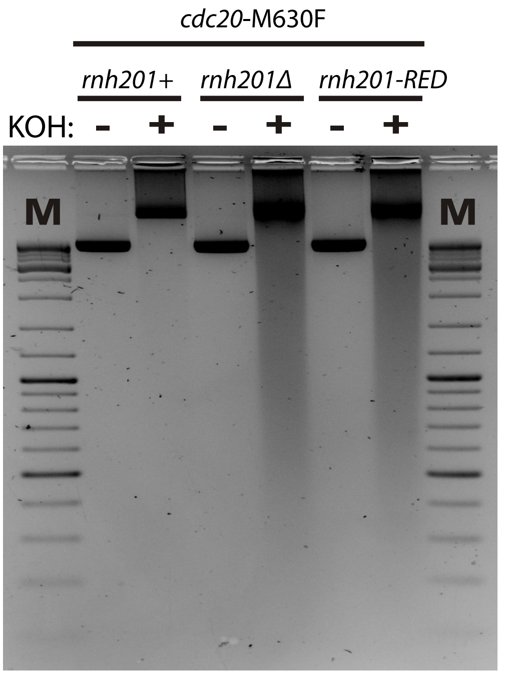
cdc20-M630F
rnh201+
rnh201Δ
rnh201-RED
KOH: - + - + - +
M
M

**c**

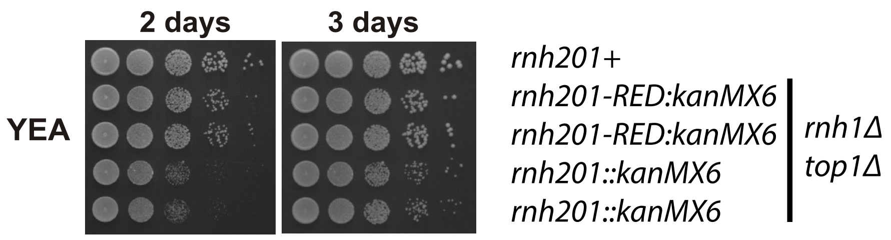
2 days
3 days
YEA
rnh201+
rnh201-RED:kanMX6
rnh201-RED:kanMX6
rnh201::kanMX6
rnh201::kanMX6
rnh1Δ
top1Δ

Fig.S8

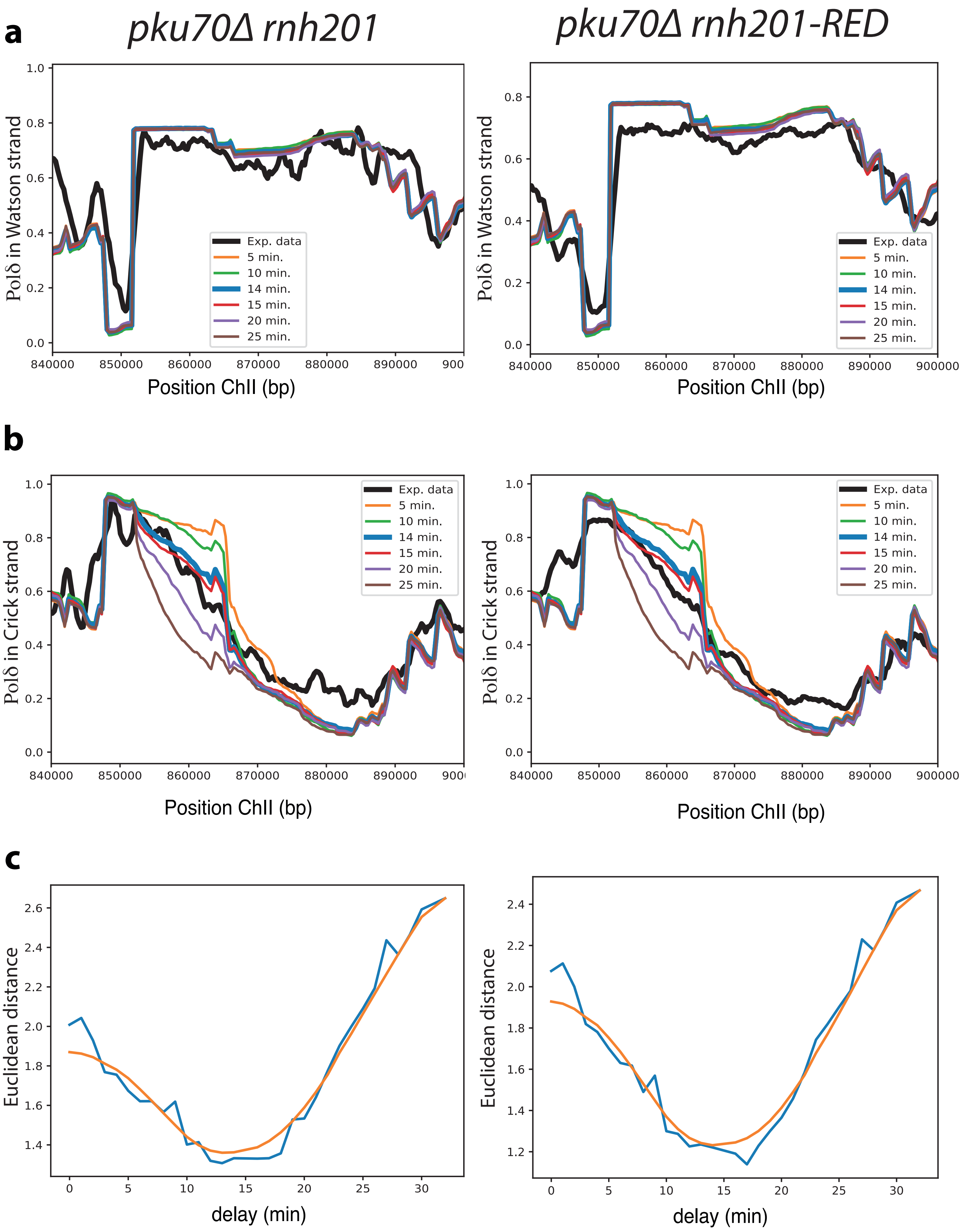
a
pku70Δ rnh201
pku70Δ rnh201-RED
Polδ in Watson strand
Exp. data
5 min.
10 min.
14 min.
15 min.
20 min.
25 min.
Position ChII (bp)
b
Polδ in Crick strand
Exp. data
5 min.
10 min.
14 min.
15 min.
20 min.
25 min.
Position ChII (bp)
c
Euclidean distance
delay (min)

Fig.S9

a

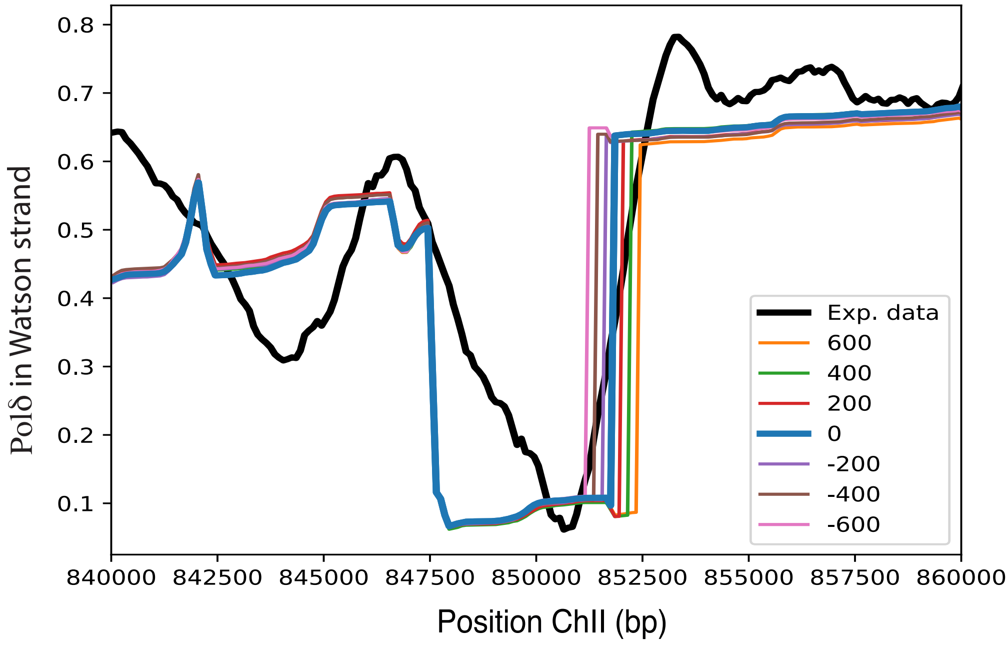
0.8
0.7
0.6
0.5
0.4
0.3
0.2
0.1
Polδ in Watson strand
840000
842500
845000
847500
850000
852500
855000
857500
860000
Position ChII (bp)
Exp. data
600
400
200
0
-200
-400
-600

b

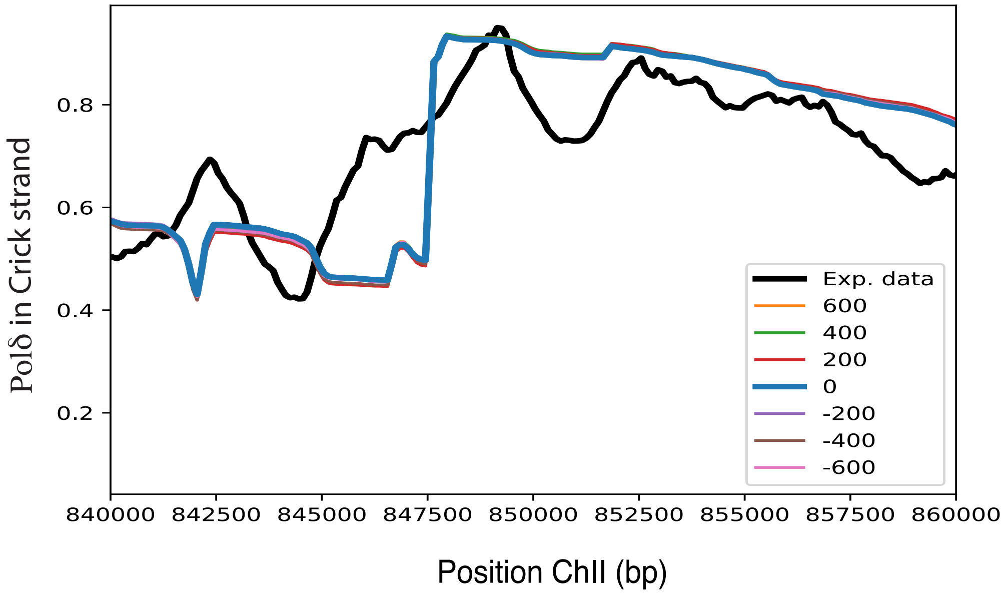
0.8
0.6
0.4
0.2
Polδ in Crick strand
840000
842500
845000
847500
850000
852500
855000
857500
860000
Position ChII (bp)
Exp. data
600
400
200
0
-200
-400
-600

c

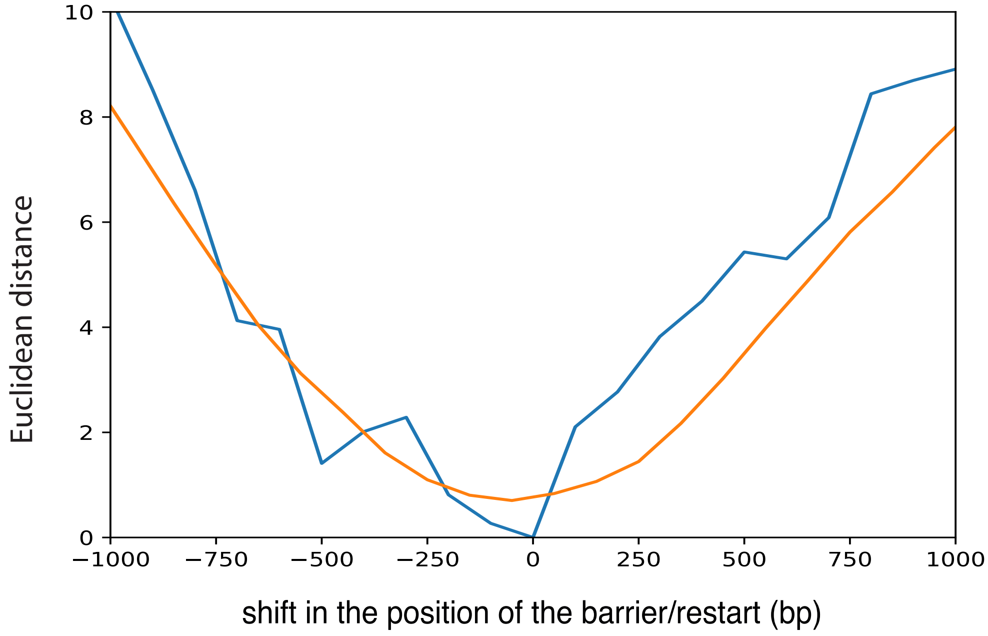
10
8
6
4
2
0
Euclidean distance
−1000
−750
−500
−250
0
250
500
750
1000
shift in the position of the barrier/restart (bp)

Supplementary Table 1. *S. pombe* strains

| Strain | Genotype | Mating type |
|---|---|---|
| 503 | *ade6-704 leu1-32 ura4-d18* | h+ |
| 995 | *cdc6-L591G ade6-704 leu1-32 ura4-d18* | h+ |
| 997 | *cdc20-M630F ade6-704 leu1-32 ura4-d18* | h+ |
| KA219 | *pol1-L850F ade6-704 leu1-32 ura4-d18* | h+ |
| KA217 | *nat:adh1-rtf1 ChII-8535:Rura-10xrRFB RTS1natural::Phleo smt0 cdc2asM17 ade6-704 leu1-32 ura4-D18* | h- |
| KA226 | *ChII-8535:Rura-10xrRFB rtf1::nat RTS1natural::Phleo smt0 cdc2asM17ade6-704 leu1-32 ura4-D18,* | h- |
| KA258 | *rpa3-GFP:kan ChII-8535:Rura-10xrRFB RTS1natural::Phleo rnh201::kan rtf1::nat smt0 rnh1::hyg cdc2asM17 ade6-704 leu1-32 ura4-D18* | h- |
| KA259 | *rpa3-GFP:kan ChII-8535:Rura-10xrRFB RTS1natural::Phleo rnh201::kan nat:adh1-rtf1 rnh1::hyg cdc2asM17 ade6-704 leu1-32 ura4-D18* | h- |
| KA261 | *rpa3-GFP:kan ChII-8535:Rura-10xrRFB RTS1natural::Phleo rtf1::NAT cdc2asM17 ade6-704 leu1-32 ura4-D18* | h- |
| KA262 | *rpa3-GFP:kan ChII-8535:Rura-10xrRFB RTS1natural::Phleo nat:adh1-rtf1 cdc2asM17 ade6-704 leu1-32 ura4-D18* | h- |
| 1144 | *rtf1::nat ChI-3220:kan ade6-704 leu1-32 ura4-d18* | h+ |
| 1145 | *rtf1::nat ChI-3220:kan ade6-704 leu1-32 ura4-d18* | h+ |
| 1146 | *rtf1::nat ChI-4740:kan ade6-704 leu1-32 ura4-d18* | h+ |
| KA2 | *rtf1::nat ChII-8535:kan ade6-704 leu1-32 ura4-d18* | h+ |
| 1158 | *rtf1::nat ChI-3220:TuraR ade6-704 leu1-32 ura4-d18* | h+ |
| 1162 | *rtf1::nat ChI-4740:TuraR ade6-704 leu1-32 ura4-d18* | h+ |
| 1166 | *rtf1::nat ChI-5234:TuraR ade6-704 leu1-32 ura4-d18* | h+ |
| KA7 | *rtf1::nat ChI-8535:TuraR ade6-704 leu1-32 ura4-d18* | h+ |
| 1170 | *rtf1::nat ChI-3220:TuraR rnh201::kan ade6-704 leu1-32 ura4-d18* | h+ |
| 1172 | *rtf1::nat ChI-4740:TuraR rnh201::kan ade6-704 leu1-32 ura4-d18* | h+ |
| 1174 | *rtf1::nat ChI-5234:TuraR rnh201::kan ade6-704 leu1-32 ura4-d18* | h+ |
| KA15 | *rtf1::nat ChI-8535:TuraR rnh201::kan ade6-704 leu1-32 ura4-d18* | h+ |
| 1177 | *ChI-3220:TuraR rnh201::kan cdc6-L591G ade6-704 leu1-32 ura4-d18* | h- |
| 1179 | *ChI-3220:TuraR rnh201::kan cdc20-M630F ade6-704 leu1-32 ura4-d18* | h- |
| 1185 | *ChI-5234:TuraR rnh201::kan cdc6-L591G ade6-704 leu1-32 ura4-d18* | h- |
| 1187 | *ChI-5234:TuraR rnh201::kan cdc20-M630F ade6-704 leu1-32 ura4-d18* | h- |
| KA31 | *ChII-8535:TuraR rnh201::kan cdc6-L591G ade6-704 leu1-32 ura4-d18* | h- |
| KA33 | *ChII-8535:TuraR rnh201::kan cdc20-M630F ade6-704 leu1-32 ura4-d18* | h- |
| KA218 | *rtf1::nat ChII-8535:kan ura4 ade6-704 leu1-32 ura4-d18* | h+ |
| KA233 | *rtf1::nat ChII-8535:kan second RTS barrier ade6-704 leu1-32 ura4-d18* | h+ |
| KA237 | *rtf1::nat ChII-8535:Rura second RTS barrier ade6-704 leu1-32 ura4-d18* | h+ |
| KA238 | *ChII-8535:Rura RTS1natural::Phleo loxP-rnh201-RED-kanR-loxM3 nat:adh1-rtf1 ade6-704 leu1-32 ura4-D18* | h- |

| | | |
|---|---|---|
| KA156 | *ChII-8535:Rura-10xrRFB RTS1natural::Phleo rnh201:kan nat:adh1-rtf1 ade6-704, leu1-32 ura4-D18* | h- |
| KA199 | *ChII-8535:Rura-10xrRFB cdc6-L591G RTS1natural::Phleo rnh201:kan nat:adh1-rtf1 ade6-704 leu1-32 ura4-D18* | h- |
| KA200 | *ChII-8535:Rura-10xrRFB cdc20-M630F RTS1natural::Phleo rnh201:kan nat:adh1-rtf1 ade6-704 leu1-32 ura4-D18* | h- |
| KA241 | *ChII-8535:Rura second RTS barrier RTS1natural:Phleo rnh201-RED:kan nat:adh1-rtf1 ade6-704 leu1-32 ura4-D18* | h- |
| KA256 | *cdc20-M630F ChII-8535:Rura second RTS barrier RTS1natural::Phleo rnh201-RED:kan nat:adh1-rtf1 ade6-704 leu1-32 ura4-D18* | h- |
| KA260 | *cdc6-L591M ChII-8535:Rura second RTS barrier RTS1natural::Phleo rnh201-RED:kan nat:adh1-rtf1 ade6-704 leu1-32 ura4-D18* | h- |
| 1048 | *rnh201::kan ade6-704 leu1-32 ura4-d18* | h+ |
| KA103 | *ChII-8535:tandemRTS1-ura4-10xrRFB rtf1::nat rnh201::kan ade6-704 leu1-32 ura4-D18* | h+ |
| SAL445 | *RTS1natural::Phleo rtf1:nat smt0* | h- |
| KA107 | *ChII-8535:tandemRTS1-ura4-10xrRFB rtf1::nat rnh201::kan RTS1natural::Phleo smt0 ade6-704 leu1-32 ura4-D18* | h- |
| KA169 | *cdc6-L591G ChII-8535:tandemRTS1-ura4-10xrRFB rnh201:kan RTS1natural::Phleo smt0 ade6-704 leu1-32 ura4-D18* | h- |
| KA170 | *cdc20-M630F ChII-8535:tandemRTS1-ura4-10xrRFB rnh201::kan RTS1natural::Phleo smt0 ade6-704 leu1-32 ura4-D18* | h- |
| KA76 | *Ku70:leu2 ChII-8535:Rura-10xrRFB RTS1natural::Phleo smt0x rnh201::kan rtf1::nat ade6-704 leu1-32 ura4d18* | h- |
| KA77 | *Ku70:leu2 ChII-8535:Rura-10xrRFB RTS1natural::Phleo smt0x rnh201::kan ade6-704 leu1-32 ura4d18* | h- |
| KA78 | *cdc6-L591G Ku70:leu2 ChII-8535:Rura-10xrRFB RTS1natural::Phleo smt0x rnh201::kan rtf1::nat ade6-704 leu1-32 ura4d18* | h- |
| KA79 | *cdc20-M630F Ku70:leu2 ChII-8535:Rura-10xrRFB RTS1natural::Phleo smt0x rnh201::kan rtf1::nat ade6-704 leu1-32 ura4d18* | h- |
| KA80 | *cdc6-L591G Ku70:leu2 ChII-8535:Rura-10xrRFB RTS1natural::Phleo smt0x rnh201::kan ade6-704 leu1-32 ura4d18* | h- |
| KA81 | *cdc20-M630F Ku70:leu2 ChII-8535:Rura-10xrRFB RTS1natural::Phleo smt0x rnh201::kan ade6-704 leu1-32 ura4d18* | h- |
| KA156 | *ChII-8535:Rura-10xrRFB RTS1natural::Phleo rnh201::kan nat:adh1-rtf1 ade6-704 leu1-32 ura4-D18* | h- |
| BAY125 | *cdc6-L591G ChII-8535:Rura-10xrRFB RTS1natural::Phleo rnh201::kan rtf1::nat ade6-704 leu1-32 ura4-D18* | h- |
| BAY123 | *cdc20-M630F ChII-8535:Rura-10xrRFB RTS1natural::Phleo rnh201::kan rtf1::nat ade6-704 leu1-32 ura4-D18* | h- |
| BAY126 | *cdc6-L591G ChII-8535:Rura-10xrRFB RTS1natural::Phleo, rnh201::kan, ade6-704 leu1-32 ura4-D18* | h- |

| | | |
|---|---|---|
| BAY124 | *cdc20-M630F ChII-8535:Rura-10xrRFB RTS1natural::Phleo rnh201::kan ade6-704 leu1-32 ura4-D18* | h- |
| KA161 | *ChII-8535:Rura-10xrRFB  rnh201-RED:kan ade6-704 leu1-32 ura4-D18* | h- |
| KA164 | *ChII-8535:Rura-10xrRFB rnh201-RED:kan RTS1natural::Phleo rtf1::nat ade6-704 leu1-32 ura4-D18* | h- |
| KA171 | *cdc6-L591G ChII-8535:Rura-10xrRFB rnh201-RED:kan RTS1natural::Phleo, smt0 ade6-704 leu1-32 ura4-D18* | h- |
| KA172 | *cdc6-L591G ChII-8535:Rura-10xrRFB rnh201-RED:kan RTS1natural::Phleo, rtf1::nat smt0 ade6-704 leu1-32 ura4-D18* | h- |
| KA173 | *cdc20-M630F ChII-8535:Rura-10xrRFB rnh201-RED:kan RTS1natural::Phleo smt0 ade6-704 leu1-32 ura4-D18* | h- |
| KA174 | *cdc20-M630F ChII-8535:Rura-10xrRFB rnh201-RED:kan RTS1natural::Phleo, rtf1:nat smt0 ade6-704 leu1-32 ura4-D18* | h- |
| KA203 | *ChII-8535:Rura-10xrRFB pol1-L850F rnh201::kan RTS1natural::Phleo ade6-704 leu1-32 ura4-D18* | h- |
| KA204 | *ChII-8535:Rura-10xrRFB pol1-L850F rnh201-RED:kan RTS1natural::Phleo, ade6-704 leu1-32 ura4-D18* | h- |
| KA205 | *ChII-8535:Rura-10xrRFB pol1-L850F rnh201::kan rtf1::nat RTS1natural::Phleo ade6-704 leu1-32 ura4-D18* | h- |
| KA206 | *ChII-8535:Rura-10xrRFB pol1-L850F rnh201-RED:kan rtf1::nat RTS1natural::Phleo ade6-704 leu1-32 ura4-D18* | h- |
| KA215 | *ChII-8535:Rura-10xrRFB rnh201-RED:kan Ku70::hyg RTS1natural::Phleo smt0 rtf1::nat ade6-704 leu1-32 ura4-D18* | h- |
| KA220 | *cdc6-L591G ChII-8535:Rura-10xrRFB rnh201-RED:kan Ku70::hyg RTS1natural::Phleo smt0 rtf1::nat ade6-704 leu1-32 ura4-D18* | h- |
| KA221 | *cdc6-L591G ChII-8535:Rura-10xrRFB rnh201-RED:kan Ku70::hyg RTS1natural::Phleo smt0 ade6-704 leu1-32 ura4-D18* | h- |
| KA222 | *cdc20-M630F ChII-8535:Rura-10xrRFB rnh201-RED:kan Ku70::hyg RTS1natural::Phleo smt0 rtf1::nat ade6-704 leu1-32 ura4-D18* | h- |
| KA223 | *cdc20-M630F ChII-8535:Rura-10xrRFB rnh201-RED:kan Ku70::hyg RTS1natural::Phleo smt0 ade6-704 leu1-32 ura4-D18* | h- |
| BAY4 | *ChI-3220:10xrRFB ade6-704 leu1-32 ura4-D18* | |
| BAY12 | *cdc6-L591G ChI-3220:10xrRFB  rnh201:kan ade6-704 leu1-32 ura4-D18* | h- |
| BAY16 | *cdc20-M630F ChI-3220:10xrRFB  rnh201:kan rtf1::nat ade6-704 leu1-32 ura4-D18* | h- |
| KA39 | *rtf1::nat ChII-8535:kan-ura4 ade6-704 leu1-32 ura4-d18* | h+ |
| KA56 | *rtf1::nat ChII-8535:kan-10xrRFB ade6-704 leu1-32 ura4-d18* | h+ |
| KA58 | *rtf1::nat ChII-8535:Rura-10xrRFB ade6-704 leu1-32 ura4-d18* | h+ |
| AW1480 | *rnh201::ura4 ura4D18 leu1-32* | h- |
| AW1563 | *rnh201-RED:kan ura4D18 leu1-32* | h- |
| AW1565 | *rnh201-RED:hyg ura4D18 leu1-32* | h- |
| AW1566 | *rnh201-RED:nat ura4D18 leu1-32* | h- |
| AW1466 | *cdc20M630F ade6-704 ura4D18 leu1-32* | h- |
| AW1405 | *cdc20M603F rnh201::kan ade6-704 leu1-32 ura4D18* | h- |

| | | |
|---|---|---|
| AW1232 | *rnh201-RED:kan cdc20-M630F* | h- |
| AW278 | | h- |
| AW1229 | *rnh201-RED:kan rnh1::nat top1::hyg* | h- |
| AW1230 | *rnh201-RED:kan rnh1::nat top1::hyg* | h+ |
| AW1198 | *rnh201::kan rnh1::nat top1::hyg* | h+ |
| AW1199 | *rnh201::kan rnh1::nat top1::hyg* | h+ |

Supplementary Table 2. Primers

| Name | Sequence |
|---|---|
| F37 qPCR -200 | ATGTGCTGTATGTTTTTTTCAGTGA |
| R38 qPCR -200 | CGATCGTTGTTAATTGGATTGGT |
| L5F qPCR +200 | AGGGCATTAAGGCTTATTTACAGA |
| L5R qPCR +200 | TCACGTTTAATTTCAAACATCCA |
| F139 qPCR+ 400 | TCGTCGGCATCTCTGCACAT |
| R140 qPCR +400 | CCTCAGCTCTAGCTGAATAGC |
| A19 qPCR +4200 | GGTTGGGGTCGTTCTGAAAC |
| A20 qPCR +4200 | ACTGACTATCACCACGCCAG |
| 461 | TATTCTCAATCCTGAACTTCTGGGTAGACATTATATAACCTATGAATTAAACA AAAGTGCTTGTTGATTTTCAATTATGACCATCGCATTCTAAAATCTATTACGC CAAGCTTGCATGGC |
| 462 | TCTACCAATGCTTCAAGTTTAAGTCACTAAATGGATTATAGAATAAGTACATC TTTAAAAAATGCTTAAGTTTTAATGATTATTTCGAGCTTCGAACCTGACGACG GCCAGTGAATTCGC |
| 463 | AACATAATACGTGGACGAGTGCAAAAAACTACCAGTTTATTTAAATAAATAA AAAAAAAATATCATAACATGTATTATCGTAATCTTTTCCTTAGACTGCTTACG CCAAGCTTGCATGGC |
| 464 | CCCTAGAGTTTTCGTTCGCAGTGCTGTGAAGGAGACTACCTGTTTCTGAAAT TTTTATAAATAGCTTTTTGTTGACGGTTATTCAGACATCGTGTGAAGAACGAC GGCCAGTGAATTCGC |
| 465 | ATAACGGTATAAAGACAATACCCATTATTATTACGTTATACCACTATAAACCA AAGTCATTTGGTAATGCCAAAACATACACTTGCCAATATTTTGAAGGTTACG CCAAGCTTGCATGGC |
| 466 | TTTCTTCTGTTTGTACTAATATGGATTCTCATAATTTACTATATTTGATAAGCA ATGAGGTTCATATTTTATTAATATACTTAATAGTGTCAAGCCCAATACGACGG CCAGTGAATTCGC |
| F3 | CCAATTAACAACGATCGTCAATGTCATTTGATGTTATTATTATACTACTCACC AAATGTTTACATCTACATAATCTAAATCTTGCTTAGTTTCCCAAGTATTACGCC AAGCTTGCATGGC |
| R4 | GGTTTCATATCATTATAGCATTTATGGTTTTTTTCATTATTGTTAAAGATCTGC ATTCAGAATTTACTTTTTTAATGATTATCTGGTTGATACATTACTCACGACGG CCAGTGAATTCGC |
| F229 | GTATTTCTCAGGACCTTTCCAGCAAACTGCGGAATCTTGGTATTTGGATAGTT GTGAAGTTTGTCACACTCAATGAGAGGGCCACGACAAAAAATTTTTGGATAC ATGTCTTGAGTCTACAGTTTACGATAAAACAGTTTTAATTGGATTTGTGATAT TGACGAAACTTTTTGAC |
| R230 | TTTCAGTTCTTCAACCTGTAATACAGAGCTTGTAAAATGTATGACCGTCAAAT ACTATGTTTAGACAAGTAAGGAAAACTCTACAAATCACCGCATAAAAATATT TTTTGATTGACGGTAATCTACTGAATTTCAAAATTTTTTTATTAAGCTACAAAT CCCACTGGCTATA |
| F241 | GTATTTCTCAGGACCTTTCCAGCAAACTGCGGAATCTTGGTATTTGGATAGTT GTGAAGTTTGTCACACTCAATGAGAGGGCCACGACAAAAAATTTTTGGATAC ATGTCTTGAGTCTACAGTTTACGATAAAACAGTTTTAATTGGATTTAAGCCCG TCTAATGAGAATTAGG |
| R242 | TTTCAGTTCTTCAACCTGTAATACAGAGCTTGTAAAATGTATGACCGTCAAAT ACTATGTTTAGACAAGTAAGGAAAACTCTACAAATCACCGCATAAAAATATT |

| | |
|---|---|
| | TTTTGATTGACGGTAATCTACTGAATTTCAAAATTTTTTTATTAATTTGTTTGTAAGGAATTGGTAGTGG |
| 264 | GATCAGAGTTTTATTTTTAGgtcgacTGGATGGCGGCGTTAGTATC |
| 265 | ATACGAAGTTATACTAGTTCgtcgacGTTTAGCTTGCCTCGTCCCC |
| F29 | AATTATCATTGCTTGAATTATACAATTAATACATTTTGCATTCATGTGCAATTCGCATTTAAGTGTAATACGAAATCTGTAGAATTTGTGGCCAAGAACCgctacaaatcccactggcta |
| R30 | GCACTAAAGAGAAAGTCCCGTTCCTTTTATTCAGTACGTTATGGGTAATACTAATAATTGCGAGAGGTTGTACAATTCTCTTTTAGTTTTTAAGAAAACTtgtgatattgacgaaacttt |
| F41 | AATTATCATTGCTTGAATTATACAATTAATACATTTTGCATTCATGTGCAATTCGCATTTAAGTGTAATACGAAATCTGTAGAATTTGTGGCCAAGAACCAGCTCATGATAATCTATTAA |
| R42 | GCACTAAAGAGAAAGTCCCGTTCCTTTTATTCAGTACGTTATGGGTAATACTAATAATTGCGAGAGGTTGTACAATTCTCTTTTAGTTTTTAAGAAAACTCTATTAGTCAGCACAGTATA |
