# Supplementary material for: Replication dynamics of recombination-dependent replication forks
Source: Nat Commun. 2021 Feb 10;12:923. doi: 10.1038/s41467-021-21198-0 (PMC7876095; doi:10.1038/s41467-021-21198-0)
Supplement: Supplementary file 4 — Description of Additional Supplementary Information [file 41467_2021_21198_MOESM4_ESM.docx]

Description of Additional Supplementary Information

Title: Supplementary Data 1.

Description: The ChIP data
